# Hepatitis B virus prevalence, vaccine uptake, and associated factors at Lodwar County Referral Hospital, Turkana County, Kenya

**DOI:** 10.11604/pamj.2026.53.52.50250

**Published:** 2026-02-03

**Authors:** Paul Gathii, Peter Karanja, Joseph Muriuki

**Affiliations:** 1 Department of Medical Laboratory Sciences, Jomo Kenyatta University of Agriculture and Technology, Nairobi, Kenya,; 2 Centre for Virus Research, Kenya Medical Research Institute, Nairobi, Kenya

**Keywords:** Hepatitis B virus, Turkana County, pastoralists, vaccination

## Abstract

**Introduction:**

hepatitis B virus (HBV) remains a major global public health concern, with disproportionate burden in marginalized populations with low vaccine uptake. Turkana County, Kenya, is characterized by a mobile nomadic population and recurrent communal conflicts, factors that may increase the risk of HBV transmission and outbreaks. This study aimed to estimate the HBV prevalence and assess vaccine uptake and factors associated with HBV infection among patients attending Lowdar Country Referral Hospital, Turkana County.

**Methods:**

this was a cross-sectional study. Data on vaccine uptake, knowledge, and practices on transmission and prevention of HBV were collected using a structured questionnaire and analysed using SPSS.

**Results:**

among the 125 study participants enrolled, 56% (n=70) were female, while 44% (n=55) were male. Hepatitis B virus prevalence was 7.2%, with over half of the participants aware of the disease, its transmission, and symptoms. Risk exposures were common, including tattoos or scarification (64%), circumcision (72%), sexual activity with low condom use (50% never used a condom), and home delivery among one-third of female participants. Hepatitis B virus vaccination coverage was low (10%), with age, marital status, number of sexual partners, and parity significantly associated with HBV infection (p<0.05).

**Conclusion:**

the prevalence of HBV was of intermediate endemicity, with moderate awareness but low vaccination coverage. Age, marital status, multiple sex partners, and parity were significantly associated with HBV infection, highlighting the need for targeted public health interventions to improve vaccine uptake among this high-risk population.

## Introduction

Globally, an estimated 296 million people were living with chronic HBV infection as of 2019, with approximately 1.5 million new infections occurring annually [1]. Hepatitis B virus is responsible for around 820,000 deaths per year, primarily due to complications such as cirrhosis and liver cancer, and most of these are in Eastern Asia and sub-Saharan Africa, where the associated complications of chronic liver disease and liver cancer are the most important health problems [2]. The global prevalence of HBV varies significantly by region, with the highest rates observed in the Western Pacific and African regions, where 6.2% and 6.1% of the adult population are chronically infected, respectively. In contrast, regions such as the Americas and Europe have relatively low prevalence rates, with less than 1% of the population infected [1].

Hepatitis B virus and other viral infections, as a causative agent for hepatitis, have a wide range of clinical presentations, ranging from an asymptomatic carrier state, which is at times self-limiting, to cases of jaundice, acute or chronic hepatitis, with progression to liver cirrhosis and hepatocellular carcinoma. Both viral factors and host immune response have been implicated in the pathogenesis and clinical outcome of HBV infection [3]. In 1991, the Global Advisory Group of the Expanded Program on Immunization (EPI) recommended hepatitis B vaccination integration into the existing national immunization programs [1,3]. This was to be achieved by 1995 in countries with an HBV carrier prevalence of 8% or higher, and by 1997 in countries with a lower. By the end of 2014, the hepatitis B vaccine had been introduced nationwide in 184 countries. The duration of protection from the HBV vaccine is estimated by WHO to be a minimum of 30 years to lifelong [4]. There has been no report of HBV outbreaks in populations with protective antibody levels. Response to HBV vaccine is influenced by age, gender, dose, quality of the antigen, number of doses, route of administration, and nutritional status of the recipient [3,4].

The disruption of health services in conflict zones and protracted humanitarian crises may increase the risk of iatrogenic infection through unsafe injection and blood transfusion [5]. Exposure to unsafe injections and procedures is a risk factor that has reported in several studies [6]. This could reflect a lack of awareness about safe injection, unsterilized equipment, and poor health infrastructure, or frequent exposure to violence and conflict requiring hospitalization and treatment, such as blood transfusions or surgeries. Some risk factors are unique to certain areas, such as barber shops amongst studies from Central Asia [6], or scarification in some areas in Africa. Some studies, such as that from Baumert and colleagues, identified multiple sex partners and unprotected sexual intercourse as risk factors associated with HBV infection. However, none of the existing literature has mentioned the potential of gender-based violence (GBV) on HBV transmission [7,8].

According to the World Health Organization [9], untreated chronic hepatitis B infection ultimately leads to liver complications such as liver cirrhosis and cancer, and hepatitis B has been causing death to over 80,000 people worldwide annually. A mere 10% of people are diagnosed with it, and 22% receive treatment [8,9]. A majority of people with chronic hepatitis B are in low- and middle-income countries (LMIC), with 20 countries accounting for 75% of infections. The World Health Assembly in 2016 adopted a goal to eliminate hepatitis B as a public health problem by 2030 [9]. The proposed study setting, Turkana, a County in the North-West of Kenya, is characterized by nomadic lifestyles, intercommunal marriage, conflicts, and is geographically remote, creating a critical public health challenge. The convergence of these and other factors may exacerbate the transmission and spread of HBV, posing significant health risks. There is a need to explore the cultural barriers and the transient nature of these communities to scale efforts to prevent and control the spread of HBV. This study aimed to estimate the prevalence of HBV infection and assess vaccine uptake and factors associated with HBV infection among patients attending Lowdar Country Referral Hospital, Turkana County.

## Methods

**Study design and settings:** this was a cross-sectional study carried out in Lodwar County Referral Hospital, Turkana County. A region on the north-west side of Kenya that is characterized by a mobile nomadic population and recurrent communal conflicts.

**Study participants:** participants were adult patients (≥18 years) seeking routine clinical services at Lodwar County Referral Hospital, Turkana County, Kenya, between May and July 2025. Eligible individuals were aged ≥18 years or older and able to provide written informed consent. Patients who were critically ill, unable to provide consent, or who declined participation were excluded. Using the hospital outpatient register as the sampling frame, we consecutively approached patients attending the outpatient department. Trained study staff explained the study objectives, procedures, potential risks, and confidentiality measures before enrollment.

**Sample size and sampling strategy:** the minimum sample size was calculated using the Cochran (1975) formulae for finite populations, assuming HBV prevalence of 8% (Kathleen *et al*.) [10], a 95% confidence level, and a 5% margin of error. This yielded a required sample size of 113 participants. A consecutive sampling strategy was employed, whereby every 5^th^ eligible client presenting to the outpatient department during the study period was invited to participate until the target sample size was exceeded. In total, 125 participants were enrolled, representing 95% of individuals approached, accounting for refusals and exclusions.

**Data collection:** data on sociodemographic (age, sex, marital status, education level), behavioural and lifestyle factors (tattoos, scarification, sexual activity, condom use, home deliveries, parity and number of sexual partners), clinical history relevant to HBV infection, vaccine uptake, knowledge on transmission and prevention of HBV and practices associated with HBV infection were collected using a structured computer-assisted personal interviewing (CAPI) questionnaire. Trained research assistants conducted face-to-face interviews and collected specimens for laboratory analysis.

**Specimen collection and laboratory analysis:** approximately 2.5ml of venous blood sample was collected from all enrolled participants in a plain tube and centrifuged to collect at least 1.25ml of serum sample for both HBV screening and ELISA (Enzyme Linked Immunosorbent Assay). Approximately 0.25ml serum sample was utilized for Rapid HBsAG testing at Lowdar County Referral Hospital Laboratory, and 1ml of serum was divided equally into 2 cryovial tubes: 0.5ml for ELISA testing and 0.5ml frozen for shipment to the KEMRI (Kenya Medical Research Institute) laboratory. A cooler box with liquid nitrogen was used with an internal thermometer to monitor temperature variation throughout the shipping process. At the Lowdar County Referral Laboratory, serum samples were tested for HBsAg using the Determine Rapid Diagnostic Test kit for HBsAg (RDTs) (Abbott Diagnostics Korea Inc, Giheung-gu, Korea) as per the manufacturer´s instructions [11]. A confirmatory test was conducted at KEMRI Laboratory, Nairobi, using HBsAg ELISA (Atlas Medical Ludwig-Erhard Rind 3 15827 Blankenfelde-Mahlow, Germany) technique as per the manufacturer's instructions and KEMRI Laboratory Standard Operating Procedure (SOP).

**Statistical analysis:** data were retrieved from the CAPI database and exported to SPSS version 28 for analysis. Descriptive statistics were used to summarize participant characteristics, with frequencies and proportions for categorical variables such as age group, marital status, and level of education. Hepatitis B virus vaccine uptake was assessed by determining participants´ vaccination status through self-report and, where available, verification using vaccination cards or official immunization cards, and presented using frequencies and proportions among vaccinated and unvaccinated individuals. Hepatitis B virus infection status was classified as positive or negative based on laboratory results. Associations between HBV infection status and participants´ sociodemographic characteristics were assessed using the Chi-square test, with comparisons made between HBV-positive and HBV-negative participants. Results were reported and interpreted based on statistically significant associations; no effect sizes or multivariable analysis were performed. A p-value of <0.05 was considered statistically significant. The overall prevalence was calculated as the proportion of participants that was seropositive for hepatitis B infection (HBsAg positive) in relation to the sample size (denominator).

**Ethical considerations:** this study was reviewed and approved by the Science, Ethics, and Research Committee of JKUAT (JKU/ISERC/02316/1412). License to conduct the study was granted by the National Commission for Science, Technology, and Innovation (NACOSTI) (235842), and permission was granted by the HMT of Lodwar County Referral Hospital. Patients were enrolled in the study after written informed consent was obtained. Personal identifiers were coded to maintain privacy.

## Results

**Sociodemographic characteristics of participants:** out of the 125 study participants enrolled, the majority were female (56%, n=70), (62%, n=77) were married, (30%, n=37) had no formal education, and (37%, n=46) were between the ages of 21 and 30 years (Table 1).

**Table 1:** sociodemographic characteristics of adults attending Lowdar County Referral Hospital, Turkana County, Kenya (n=125), May - July 2025

Characteristic		n	%
**Age band**	< 20 years	30	24.0
	21-30 years	46	36.8
	31-40 years	30	24.0
	41-50 years	8	6.4
	> 50 years	11	8.8
**Sex**	Female	70	56.0
	Male	55	44.0
**Marital status**	Cohabiting	2	1.6
	Married	77	61.6
	Single	46	36.8
**Number of children**	None	34	27.2
	One	20	16.0
	Two	21	16.8
	Three	9	7.2
	More than three	41	32.8
**Highest level of education**	College education	13	10.4
	No education	37	29.6
	Primary education	36	28.8
	Secondary education	35	28.0
	University education	4	3.2
**Occupation**	Business/self-employed	35	28.0
	Casual labourer	39	31.2
	Farmer	5	4.0
	Herdsman	13	10.4
	Housewife	28	22.4
	Medical personnel	3	2.4
	Security personnel	1	0.8
	Teacher (University)	1	0.8
**Income band**	< Ksh 5000	46	36.8
	Between Ksh 5001-10000	27	21.6
	Between Ksh 10001-20000	11	8.8
	Between Ksh 20001-30000	19	15.2
	Between Ksh 30001-40000	5	4.0
	> Ksh 40001	17	13.6
**Religion**	Christian	83	66.4
	Muslim	3	2.4
	Others	19	15.2
	Protestant	20	16.0

**Prevalence and factors associated with HBV infection:** the overall prevalence of HBV infection among the study participants was 7.2% (9/125, 95% CI: 2.7-11.7). Table 2 presents the association between sociodemographic characteristics and HBV Infection among the study participants. The study findings revealed that there was a statistically significant association between age and hepatitis B infection (χ^2^= 28.542, p<0.001). Infections were more common among participants aged 31-40 years (4 cases) and 41-50 years (4 cases), while only one case was observed among the 21-30 years group. No infections were recorded in participants below 20 years or above 50 years. There was no significant association observed between gender and hepatitis B infection (χ^2^= 2.022, p = 0.155) despite slightly more infections being recorded among males (6 cases) compared to females (3 cases). Marital status was found to be significantly associated with hepatitis B infection (χ^2^= 6.046, p = 0.049). The majority of the HBV infections occurred among married participants (9 cases), while no cases were reported among single individuals or among those cohabiting.

**Table 2:** association between sociodemographic characteristics and HBV infection among adults attending Lodwar County Referral Hospital, Turkana County

Characteristic		Rapid hepatitis B surface antigen (HBsAg)		Total	Chi-square	P-value
		Non-reactive	Reactive			
**Age group**	< 20 years	30	0	30	28.542	<0.001
	21-30 years	45	1	46		
	31-40 years	26	4	30		
	41-50 years	4	4	8		
	> 50 years	11	0	11		
**Sex**	Female	67	3	70	2.022	0.155
	Male	49	6	55		
**Marital status**	Cohabiting	2	0	2	6.046	0.049
	Married	68	9	77		
	Single	46	0	46		
**Number of children**	None	34	0	34	15.327	0.004
	One	20	0	20		
	Two	21	0	21		
	Three	8	1	9		
	More than three	33	8	41		
**Highest level of education**	College education	11	2	13	3.505	0.477
	No education	33	4	37		
	Primary education	35	1	36		
	Secondary education	33	2	35		
	University education	4	0	4		
**Occupation**	Business/self-employed	33	2	35	2.209	0.947
	Casual labourer	36	3	39		
	Farmer	5	0	5		
	Herdsman	11	2	13		
	Housewife	26	2	28		
	Medical personnel	3	0	3		
	Security personnel	1	0	1		
	Teacher (University)	1	0	1		
**Income band**	< Ksh 5000	43	3	46	2.750	0.739
	Between Ksh 5001-10000	25	2	27		
	Between Ksh 10001-20000	11	0	11		
	Between Ksh 20001-30000	18	1	19		
	Between Ksh 30001-40000	4	1	5		
	> Ksh 40001	15	2	17		
**Religion**	Christian	78	5	83	2.542	0.637
	Muslim	3	0	3		
	Others	17	2	19		
	Protestant	18	2	20		

The number of children was significantly associated with hepatitis B infection (χ^2^= 15.327, p = 0.004). Participants with more than three children had the highest number of infections (8 cases), while no infections were observed among those with fewer children. Education level was significantly associated with hepatitis B infection (χ^2^= 3.505, p = 0.477) as the cases were mostly observed among individuals with no formal education (4 cases), college education (2 cases), primary (1 case), and secondary (2 cases) level of education. Infection was significantly associated with occupation, including self-employment (2 cases), casual labourers (3 cases), herdsmen (2 cases), and housewives (2 cases) (χ^2^= 2.209, p = 0.947). Income level and religion showed no significant association with hepatitis B infection (χ^2^= 2.750, p = 0.739; χ^2^= 2.542, p = 0.637).

**Knowledge of hepatitis B:** the participants´ knowledge regarding hepatitis B is presented in Table 3. More than half of the respondents (52.8%) reported having heard about hepatitis B infections, while 47.2% had never heard of the disease. When asked about the causes of hepatitis, the responses were diverse and often inaccurate; 28.0% correctly identified viruses as the cause, others attributed the disease to factors such as alcohol (10.4%), bacteria (4.8%), and fungi (1.6%). Notably, 38.4% selected “other causes.” Sixty percent correctly acknowledged that HBV is contagious, while 39.2% believed otherwise. Regarding long-term complications, slightly more than half (54.4%) knew that HBV could cause liver cancer. In terms of populations at risk, over half of respondents (51.2%) were aware that both adults and children could be infected, while 42.4% associated the disease primarily with adults. Personal experience with hepatitis, including HBV, was minimal, with 98.4% reporting that they had never suffered from the disease and 1.6% indicating past infection. Nearly half of the respondents (48.0%) knew someone who had suffered or was suffering from hepatitis. When asked about symptoms, the majority (52.0%) identified jaundice-yellow coloration of the skin and mucous membranes as the main sign. Others mentioned stomachache (24.0%), fatigue (4.8%), and nausea/vomiting (1.6%). Less than 2% were aware that HBV disease may have a combination of multiple symptoms.

**Table 3:** knowledge on hepatitis B infection among study participants in Lodwar County Referral Hospital, Turkana County, Kenya (n=125)

Questions		n	%
Have you ever heard about hepatitis B infections?	No	59	47.2
	Yes	66	52.8
If YES, do you know the causes of hepatitis?	Alcohol	13	10.4
	Alcohol, malaria	1	0.8
	Alcohol, others	8	6.4
	Bacteria	6	4.8
	Bacteria, alcohol	2	1.6
	Bacteria, others	1	0.8
	Fungal	2	1.6
	Malaria, others	1	0.8
	Others	48	38.4
	Virus or viruses	35	28.0
	Virus or viruses, alcohol	5	4.0
	Virus or viruses, alcohol or other	1	0.8
	Virus or viruses, bacteria	1	0.8
Is hepatitis a contagious disease?	No	49	39.2
	Yes	76	60.8
Does the hepatitis B virus cause cancer of the liver?	No	57	45.6
	Yes	68	54.4
Who are most often infected with the hepatitis B virus?	Adults	53	42.4
	Both	64	51.2
	Children	2	1.6
	Don’t know	6	4.8
Have you ever suffered from hepatitis?	No	123	98.4
	Yes	2	1.6
Do you know anyone who is suffering/who suffered from hepatitis?	No	65	52.0
	Yes	60	48.0
major signs of hepatitis B virus infection	Fatigue	6	4.8
	Fatigue, stomach ache	1	0.8
	Nausea, vomiting	2	1.6
	Nausea, vomiting, stomach ache	2	1.6
	Stomach ache	30	24.0
	Yellow coloration of the skin and mucous membranes	65	52.0
	Yellow colouration of the skin and mucous membranes, nausea, vomiting	1	0.8
	Yellow colouration of the skin and mucous membranes, nausea, vomiting, fatigue, stomachache	2	1.6
	Yellow colouration of the skin and mucous membranes, stomach ache	16	12.8
Can environmental sanitation reduce the transmission of hepatitis?	No	14	11.2
	Yes	111	88.8
Does a mosquito transmit hepatitis B virus infection?	No	115	92.0
	Yes	10	8.0
Is it possible for someone who suffers/suffered from hepatitis to get married and have children?	No	14	11.2
	Yes	111	88.8
Is the hepatitis B virus transmitted through sex?	No	58	46.4
	Yes	67	53.6
Can the hepatitis B virus be passed from mother to child during birth?"	No	32	25.6
	Yes	93	74.4
Can the hepatitis B virus be transmitted through blood and blood products?	No	33	26.4
	Yes	92	73.6
Can the hepatitis B virus be transmitted at the barbershop? Ear piercing? Nose piercing?	No	67	53.6
	Yes	58	46.4
Can the spread of the hepatitis B virus be prevented?	No	15	12.0
	Yes	110	88.0
Who told you about hepatitis B?	Community health promoters	71	56.8
	health care providers	47	37.6

The majority (88.8%) of the respondents recognized the role of environmental sanitation in reducing transmission, with nearly all (92.0%) correctly rejecting mosquito bites as a transmission route. More than three-quarters (74.4%) of the respondents were aware of mother-to-child transmission. Similarly, 73.6% were aware of blood and blood product transmission, while over a quarter (26.4%) were unaware. Sexual transmission was correctly identified by the majority, though 46.4% did not recognize it as a route. Only 46.4% associated HBV with risks from barbering, ear piercing, or nose piercing. The majority (88.0%) believed HBV spread can be prevented, and a similar proportion (88.8%) affirmed that someone with HBV can still marry and have children. Community health promoters emerged as the most common source of information (56.8%), followed by health care providers (37.6%).

**Practices associated with hepatitis B infection:** Table 4 presents the prevalence of practices related to hepatitis B virus infection in the study population. Circumcision was reported as a common practice among the community, with 68.8% affirming its prevalence. Among those circumcised, the majority (72.0%) underwent the procedure at hospitals, while 1.6% reported being circumcised by traditional practitioners. Risk-related practices such as tattoos, ear piercings, and scarification were reported by 64.0% of the respondents, while 36.0% had never engaged in such practices. Most respondents (84.8%) reported having a sexual partner. Condom use was low, with 58.4% reporting never using one. Among the users, consistent condom use was rare (4.8%), while 24.0% used them occasionally.

**Table 4:** respondents' practices on Hepatitis B infection in Lodwar County Referral Hospital, Turkana County, Kenya (n=125)

Question		n	%
Is circumcision a common practice among the members of your community?	No	39	31.2
	Yes	86	68.8
If yes, where is the procedure done?	N/A	33	26.4
	Hospital by a healthcare worker	90	72.0
	Traditional circumciser	2	1.6
Do you have tattoos or ear piercings, or any other body scarification?	No	45	36.0
	Yes	80	64.0
Do you have a sexual partner?	No	19	15.2
	Yes	106	84.8
If yes, how many partners do you have?	0	19	15.2
	1	85	68.0
	2	6	4.8
	> 3	15	12
Have you ever used a condom?	No	86	68.8
	Yes	39	31.2
How frequently do you use a condom during sexual intercourse?	Always	6	4.8
	Never	73	58.4
	Often	8	6.4
	Rarely	8	6.4
	Sometimes	30	24.0
Where do you always go for hair shaving?	At home	55	44.0
	Barber shop	70	56.0
Have you ever been transfused with blood?	No	83	66.4
	Yes	42	33.6
Have you ever sustained an injury from animal handling?	No	60	48.0
	Yes	65	52.0
If yes to the previous question, did you visit a health facility for treatment?	NA	37	29.6
	No	37	29.6
	Yes	51	40.8
Where do women usually give birth?	At home	43	34.4
	At the hospital	82	65.6
Where was you child (children) born?	At home	41	32.8
	At the hospital	63	50.4
	At the hospital, at home	21	16.8
Have you been vaccinated with the hepatitis B vaccine?	No	112	89.6
	Yes	13	10.4
Are your children vaccinated against hepatitis B?		44	35.2
	NA	7	5.6
	No	72	57.6
	Yes	2	1.6
Is there a cure for hepatitis B?	No	20	16.0
	Yes	105	84.0
Is hepatitis B curable?	No	20	16.0
	Yes	105	84.0
Are there traditional herbs for treatment of hepatitis B?	No	45	36.0
	Yes	80	64.0
Have you been vaccinated against the hepatitis B virus?	No	112	89.6
	Yes	13	10.4
If vaccinated, how many times were you vaccinated?		110	88.0
	Can’t remember	1	0.8
	Once	2	1.6
	Thrice	8	6.4
	Twice	4	3.2
Does the distance to the nearest health facility affect your health services utilization?	No	22	17.6
	Yes	103	82.4
Does your local healthcare facility have enough and available vaccines (if experienced stock-outs)?	No	89	71.2
	Yes	36	28.8
Does the staff attitude in your local healthcare facility affect your health services utilization?	No	62	49.6
	Yes	63	50.4
Does the time the health facilities are open affect your health services utilization?	No	60	48.0
	Yes	65	52.0
Does the health care facility charge you for vaccine administration?	No	46	36.8
	Yes	79	63.2

Hair cut was mostly done in barber shops (56.0%), while 44.0% did it from home. Occupational risks were also evident: 52.0% reported sustaining injuries during animal handling. Among those injured, only 40.8% sought medical care, while 29.6% did not, and 29.6% did not respond (NA). Hospital-based delivery was more common (65.6%) compared to home deliveries (34.4%). Similarly, 50.4% of respondents reported their children were born in hospitals, while 32.8% were home births, and 16.8% had mixed experiences. Despite this relatively high hospital delivery rate, vaccination coverage for children was very low. Less than 2% reported that their children were vaccinated against HBV, while 57.6% indicated no vaccination (Table 4).

**Hepatitis B vaccine uptake:** hepatitis B vaccination coverage among adults was low, with only 10.4% reporting being vaccinated. The vaccination timeline shows sporadic uptake, with a few individuals receiving vaccines between 2019 and 2024, but the majority (88.0%) reporting no vaccination. Among those vaccinated, 6.4% completed the recommended three doses, while smaller proportions received one or two doses, and some could not recall the exact doses received. Most respondents (84.0%) believed that hepatitis B is curable, with 64.0% reporting they believe in traditional herbs for treatment. Barriers to healthcare access were evident, as attested by the majority (82.4%) who reported that distance to health facilities affected their utilization of services, while 71.2% indicated that local facilities experienced stock-outs of vaccines. Staff attitude (50.4%) and limited facility operating hours (52.0%) were also cited as constraints. Furthermore, the majority (63.2%) of the respondents asserted that they were charged for vaccine administration (Table 4). Table 5 presents the association between various knowledge-related constructs and hepatitis B Virus (HBV) infection. Awareness of hepatitis B virus infection did not differ significantly by HBV status (χ^2^= 0.030, p = 0.864). Hepatitis B virus positivity was similar among participants who had heard about hepatitis B (7.6%, 5/66) and those who had not (6.8%, 4/59).

**Table 5:** association between knowledge on HBV and infection status among adults attending Lodwar County Referral Hospital, Turkana County

Characteristic		Rapid hepatitis B surface antigen (HBsAg)		Total	Chi-square	P-value
		Non-reactive	Reactive			
Have you ever heard about hepatitis B infections?	No	55	4	59	0.030	0.864
	Yes	61	5	66		
If YES, do you know the causes of hepatitis?		1	0	1	4.627	0.983
	Alcohol	13	0	13		
	Alcohol, malaria	1	0	1		
	Alcohol others	8	0	8		
	Bacteria	5	1	6		
	Bacteria, alcohol	2	0	2		
	Bacteria, and others	1	0	1		
	Fungal	2	0	2		
	Malaria and others	1	0	1		
	Others	44	4	48		
	Viruses	32	3	35		
	Viruses, alcohol	4	1	5		
	viruses, alcohol, others	1	0	1		
	Viruses and bacteria	1	0	1		
Is hepatitis a contagious disease?	No	45	4	49	0.112	0.738
	Yes	71	5	76		
Does the hepatitis B virus cause cancer of the liver?	No	53	4	57	8.942	0.005
	Yes	63	5	68		
Who is most often infected with the hepatitis B virus?	Adults	49	4	53	1.056	0.788
	Both	60	4	64		
	Children	2	0	2		
	Don’t know	5	1	6		
Have you ever suffered from hepatitis?	No	115	8	123	5.572	0.018
	Yes	1	1	2		
Do you know anyone who is suffering/who suffered from hepatitis?	No	62	3	65	1.354	0.245
	Yes	54	6	60		
Can environmental sanitation reduce the transmission of hepatitis?	No	14	0	14	1.223	0.269
	Yes	102	9	111		
Does a mosquito transmit hepatitis B virus infection?	No	107	8	115	0.128	0.721
	Yes	9	1	10		
Is it possible for someone who suffers/suffered from hepatitis to get married and have children?	No	14	0	14	1.223	0.269
	Yes	102	9	111		
Is the hepatitis B virus transmitted through sex?	No	54	4	58	0.015	0.903
	Yes	62	5	67		
Can the hepatitis B virus be passed from mother to child during birth?"	No	30	2	32	0.058	0.810
	Yes	86	7	93		
Can the hepatitis B virus be transmitted through blood and blood products?	No	30	3	33	0.240	0.624
	Yes	86	6	92		
Can the hepatitis B virus be transmitted at the barber shop? Ear piercing? Nose piercing?	No	64	3	67	1.602	0.206
	Yes	52	6	58		
Can the spread of the hepatitis B virus be prevented?	No	14	1	15	0.007	0.932
	Yes	102	8	110		
Who told you about hepatitis B?	None	6	1	7	1.302	0.521
	Community health promoters	65	6	71		
	Health care providers	45	2	47		

Respondents´ knowledge of the causes of hepatitis was not significantly associated with HBV infection status (χ^2^= 4.627, p=0.983). Among those who correctly identified viruses as the causative agent (35 respondents), three were positive for HBV. There was no significant association between perceiving hepatitis as contagious and infection status (χ^2^= 0.112, p = 0.738). Similarly, on HBV as a cause of liver cancer, infections were more common among those who did not know HBV can cause liver cancer (4 cases) compared to those who did (5 cases), although the association was not statistically significant (χ^2^= 8.942, p = 0.005).

## Discussion

Adequate community knowledge, safe practices, and uptake for the hepatitis B virus (HBV) vaccine remain a major public health milestone in the transmission and prevention of HBV. This study aimed to estimate the prevalence of HBV infection and assess vaccine uptake and factors associated with HBV infection among patients attending Lowdar Country Referral Hospital, Turkana County. Overall, our findings report an intermediate HBV prevalence, demonstrate partial awareness of HBV causes and transmission, persistent misconceptions, low vaccine coverage, and behavioral factors such as low condom use and multiple sexual partners as factors associated with HBV infection. Our findings highlight the knowledge-practice gap and point toward structural and behavioral barriers that sustain HBV transmission. The HBV prevalence observed in this study is consistent with intermediate to high endemicity levels reported in similar populations in sub-Saharan Africa. General population studies in many sub-Saharan African settings often report HBV prevalence ranging between 5% to 10%, or sometimes higher, depending on vaccination coverage and risk behaviours [12-14].

Respondents correctly identified HBV as viral in origin, with more than one-third attributing it to “other causes” and smaller proportions linking it to alcohol consumption or lifestyle factors. This aligns with recent studies in sub-Saharan Africa showing that partial knowledge is widespread but insufficient to foster protective practices [12,13]. Studies in Kenya confirm that while general awareness of HBV exists, significant misconceptions about etiology and risk persist, particularly regarding sexual and parenteral transmission [14]. Such findings support the argument that educational campaigns must not only increase awareness but also target specific misinformation. Health behavior models suggest that accurate knowledge must be coupled with self-efficacy and structural support to influence health-seeking behavior [15]. In this context, community health promoters, already identified as the main source of information, should be equipped with standardized, evidence-based messaging to address misconceptions around barbering, body modification, and mother-to-child transmission.

Notably, our study findings on vaccination coverage were extremely low, with a small proportion of children and adults having received the HBV vaccine, despite relatively high facility delivery rates (65.6%). This suggests missed opportunities for administering the HBV birth dose. According to the World Health Organization [1], timely administration of the birth dose within 24 hours prevents up to 90% of perinatal HBV infections. However, recent reviews indicate that Kenya and many African countries still face logistical, policy, and cold-chain challenges in rolling out universal birth-dose vaccination [13,16]. The implication is that programmatic interventions should prioritize strengthening health system capacity to deliver the birth dose universally, rather than selectively targeting infants of HBV-positive mothers. Strengthening immunization records, ensuring vaccine availability in maternity wards, and monitoring coverage through routine health information systems would address the gap highlighted in this study.

Our study findings further revealed that sexual behaviors, including the number of partners and inconsistent condom use, were significantly associated with HBV infection. This is consistent with recent seroprevalence studies in East Africa, which show that multiple sexual partners and low condom use are major drivers of HBV transmission [17]. Although condom use showed a borderline protective effect in the present study, global evidence supports its role in reducing HBV transmission alongside other sexually transmitted infections [18]. Most of the participants in this study recognized the role of environmental sanitation in reducing transmission. Likewise, nearly all the participants correctly rejected mosquito bite as a transmission route, and misconceptions persisted in other areas. While more than three-quarters understood mother-to-child transmission, with the majority being aware of blood and blood product transmission. Sexual transmission was correctly identified by more than half of the participants, with a similar proportion associating HBV with risks from barbering, ear piercing, or nose piercing, despite these being well-documented risk factors. The low recognition of sexual transmission in this population underscores a neglected pathway in community messaging. Integrating HBV prevention into sexual and reproductive health programs, including condom promotion and partner screening, can strengthen public health interventions.

Three-quarters of the respondents in this study reported having body modifications, with less than half recognizing their HBV risk. Studies in sub-Saharan Africa consistently identify tattooing, piercing, and barbering with unsterilized equipment as important non-clinical risk factors for HBV transmission [17]. This suggests that risk reduction strategies need to include regulatory measures for informal service providers, coupled with public education on the importance of sterilization and safe practices. The clustering of infections among individuals aged 31-50 years, and the associations with marital status and number of children, suggest both cohort and behavioral effects. Older adults may not have benefited from infant vaccination, while married individuals with larger families may have higher lifetime exposure risks. National studies confirm that older cohorts in Kenya exhibit higher HBV prevalence than younger age groups [14]. These findings point to the need for catch-up vaccination campaigns targeting reproductive-age adults, alongside family-based screening and care strategies [9].

There was a moderate awareness level within the study population, and this suggested that information dissemination efforts have reached some communities, but knowledge gaps remain substantial. Close to a third correctly identified viruses as the cause; others attributed the HBV disease to factors such as alcohol, bacteria, and fungi. Notably, nearly half of the respondents selected “other causes,” which may reflect misinformation or lack of clarity, considering that hepatitis can be caused by different factors. Three-quarters correctly acknowledged that HBV is contagious, while more than a third believed otherwise, reflecting partial understanding of transmission dynamics. Regarding long-term complications, slightly more than half of the study participants knew that the hepatitis B virus could cause liver cancer. Over half of the respondents believed that both adults and children could be infected, while less than half associated the disease primarily with adults. This indicated some degree of awareness of universal susceptibility. The majority identified jaundice as the main manifestation in people presenting with HBV disease, which was consistent with similar studies done in other parts of the world, particularly LMIC [1,18].

**Limitations:** the study was conducted in hospital settings, and the participants may not be representative of the broader community population; therefore, our findings may not be generalizable beyond the study site. Additionally, the study primarily focused on those who could access hospital settings, overlooking other community members who engage in traditional or herbal treatment.

## Conclusion

In this hospital-based study, the observed prevalence of HBV corresponded to an intermediate level of endemicity within the study population. Awareness and knowledge of hepatitis B among participants were moderate, with gaps and misconceptions related to causes, transmission, and prevention. Despite some awareness and access to healthcare services, vaccination coverage was low and protective practices, including consistent condom use, were suboptimal among participants in this study. HBV infection was associated with selected sociodemographic and behavioural characteristics, including age, marital status, number of children, and sexual behaviour, within the study population. These findings highlight the need for strengthened HBV health education, improved vaccination uptake, and integration of HBV prevention services within routine hospital-based care. Future studies incorporating both hospital and community populations, as well as mixed-methods approaches, may help to contextualize these findings and support broader interpretation.

### What is known about this topic

- Hepatitis B virus remains highly prevalent globally, particularly in low-resource and marginalized populations, and is a leading cause of chronic liver disease and mortality;
- Despite the availability of effective vaccines, coverage is often low in hard-to-reach or mobile populations, sustaining the risk of infection and transmission.

### What this study adds

- We found intermediate endemicity of HBV in our study population;
- Our study demonstrates that awareness of HBV among the study participants is moderate, and vaccination coverage is low;
- We found that age, marital status, multiple sexual partners, and parity were significantly associated with HBV infection.
